# Fluorescence angiography after vascular ligation to make the ileo-anal pouch reach

**DOI:** 10.1007/s10151-021-02447-2

**Published:** 2021-05-15

**Authors:** J. J. Joosten, M. A. Reijntjes, M. D. Slooter, M. Duijvestein, C. J. Buskens, W. A. Bemelman, R. Hompes

**Affiliations:** 1 Department of Surgery, Amsterdam University Medical Centers (UMC), Location Academic Medical Centre (AMC), University of Amsterdam, Postbox 22660, 1100 DD Amsterdam, The Netherlands; 2 Department of Gastroenterology and Hepatology, Amsterdam University Medical Centers, Amsterdam Gastroenterology Endocrinology Metabolism (AGEM) Research Institute, University of Amsterdam, Amsterdam, The Netherlands

**Keywords:** Fluorescence angiography (FA), Indocyanine green (ICG), Ileal pouch-anal anastomosis (IPAA), Vascular ligation, Anastomotic leakage

## Abstract

The two most essential technical aspects of any gastrointestinal anastomosis are adequate perfusion and sufficient reach. For ileal pouch-anal anastomosis (IPAA), a trade-off exists between these two factors, as lengthening manoeuvers to avoid tension may require vascular ligation. In this technical note, we describe two cases in which we used indocyanine green (ICG) fluorescence angiography (FA) to assess perfusion of the pouch after vascular ligation to acquire sufficient reach. In both cases, FA allowed us to distinguish better between an arterial inflow problem and venous congestion than white light assessment. Both pouches remained viable and no anastomotic leakage occurred. Our results indicate that ICG FA is of great value after vascular ligation to obtain reach during IPAA.

## Introduction

Restorative proctocolectomy with ileal pouch-anal anastomosis (IPAA) is the surgical treatment of choice in patients with familial adenomatous polyposis (FAP), ulcerative colitis (UC) and well-selected patients with Crohn’s colitis [1]. An Achilles’ heel of pouch surgery is anastomotic leakage with a reported incidence of up to 15% [2, 3].

The key in anastomotic healing is traction-free anastomosis that is well-vascularized [4]. To acquire length in IPAA, routine lengthening measures are taken that include mobilization of the mesenteric root up to the duodenum and bilateral transverse peritoneal incisions approximately every 3 cm. In case adequate length cannot be obtained by these routine manoeuvers, tailored lengthening measures, involving vascular ligation, might be required. The ileocolic trunk (if still present) or interconnecting terminal ileal branches can be sacrificed to gain additional ileal length for the pouch to reach [5]. However, by ligating part of the arterial supply and venous drainage of the ileum, pouch perfusion can be impaired.

Intraoperative fluorescence angiography (FA) using indocyanine green (ICG) is nowadays widely applied to assess colonic vascular perfusion and could contribute to the prevention of anastomotic leakage secondary to perfusion restriction [6, 7]. So far, little is known about the role of FA in IPAA. Spinelli et al. [8] have shown that the postoperative anastomotic leakage rate after IPAA in their FA group was similar to that in a non-FA group, even though ligation of the ileocolic artery was performed more often within the FA group than non-FA group (47% versus 16%).

In this technical note, we demonstrate the potential value of FA in decision making after ligation of vessels to obtain adequate length for a tension-free anastomosis in two cases.

## Technique

As a standard technique, the completion proctectomy and IPAA were performed by a combined abdominal and transanal minimally invasive approach. After adhesiolysis, the root of the small bowel mesentery was mobilized to the level of the duodenum followed by multiple transverse peritoneal incisions. After completion of these steps, the terminal ileum was exteriorised through a Pfannenstiel incision or the stoma site. The yardstick for sufficient length was when the apex of the J-pouch reached 1–2 cm below the pubic bone. If these routine lengthening measures were insufficient, the ileocolic trunk and/or interconnecting terminal ileal branches were ligated. After sufficient length was achieved, the J-pouch was constructed by a side-to-side ileal anastomosis using a linear stapler. The anvil was placed at the apex of the J-pouch, after which the redundant blind loop was resected using a linear stapler and reinforced by sutures.

After visual inspection of the pouch in white light, ICG (VERDYE, Diagnostic Green dissolved in 10 ml sterile water) 0.1 mg/kg/bolus was injected intravenously for FA. Imaging was performed by a laparoscopic 1688 AIM camera (Stryker, Kalamazoo, MI, U.S.A.). After assessment by FA and necessary changes based on FA findings, a double purse-string single stapled ileo-anal anastomosis was fashioned followed by routine suture reinforcement of the anastomosis. A deverting ileostomy was only created in case of technical problems (e.g. staple misfire, positive reverse leak test, insufficient reach) or impression of suboptimal perfusion on FA. The ileo-anal anastomosis was assessed endoscopically within 2 weeks after surgery if a diverting ileostomy had been fashioned.

**Fig. 1:**
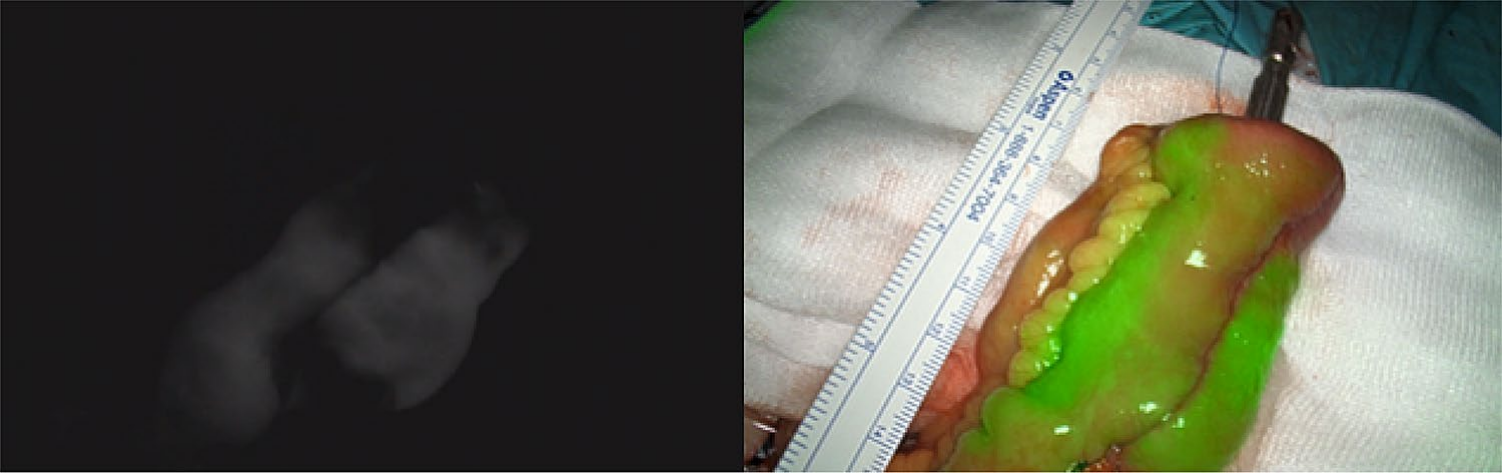
Intraoperative fluorescent and overlay image of pouch showing poor fluorescent enhancement of the apex of the pouch

**Fig. 2:**
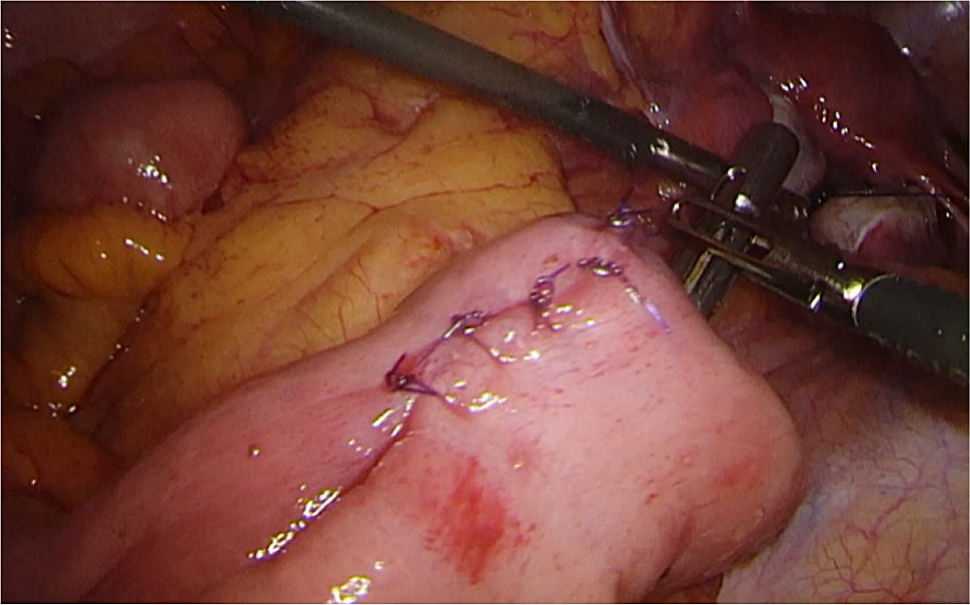
Suture reinforcement of the apex of the pouch was performed after the fluorescent images

**Fig. 3:**
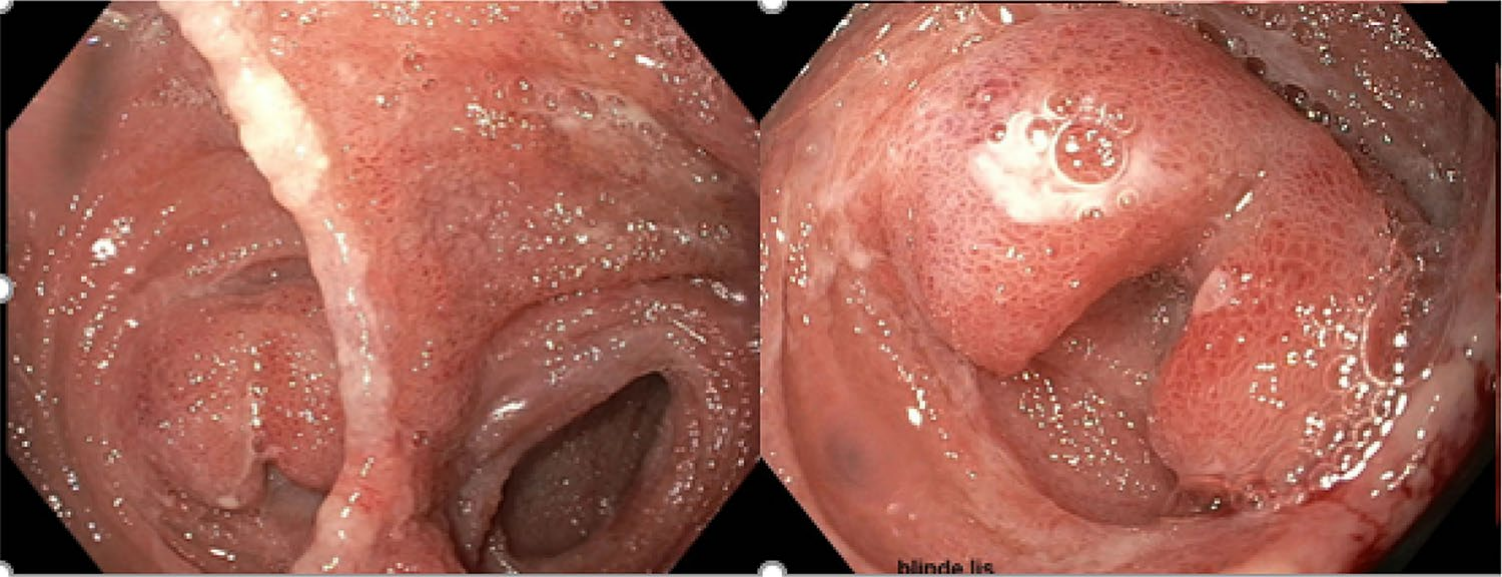
Endoscopy images of the pouch 2 weeks postoperatively showing a vital apex of the pouch and pouch body

The first case was a 23-year-old female patient diagnosed with UC. With the disease not responding to medical therapy, she underwent a subtotal colectomy and ileostomy procedure. During this procedure, the ileocolic artery was already ligated. After a 4-month convalescence period, she was referred to our unit for the second stage of a modified two-stage IPAA procedure. Intraoperatively, there was insufficient length after applying the routine lengthening measures. To gain additional length, interconnecting terminal ileal branches were ligated. After pouch construction, there was the impression of impaired perfusion of the apex of the pouch (Fig. 1) that was not observed by white light assessment. This area was reinforced by separate sutures PDS^®^ 4.0 (Ethicon, Germany) (Fig. 2). These findings also led to a further change in routine management, namely the creation of a diverting ileostomy. Postoperative endoscopy of the pouch 2 weeks after the procedure showed a well-vascularized apex of the pouch, pouch body (Fig. 3) and intact anastomosis. Stoma reversal 6 weeks later was planned.

**Fig. 4:**
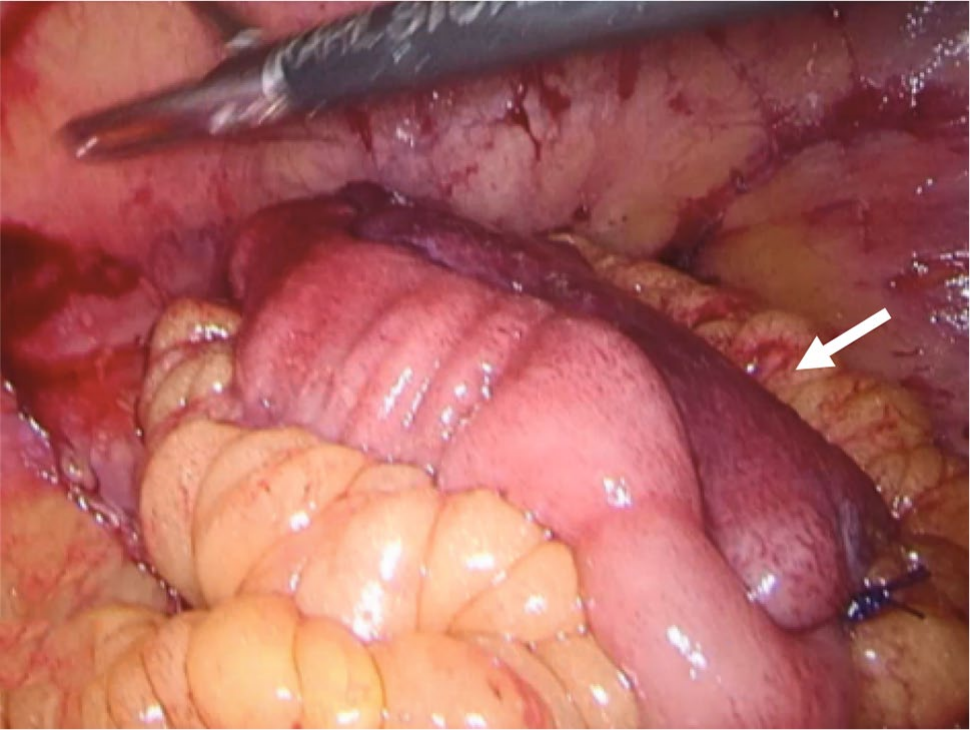
Intraoperative discoloring of the afferent loop of the pouch

**Fig. 5:**
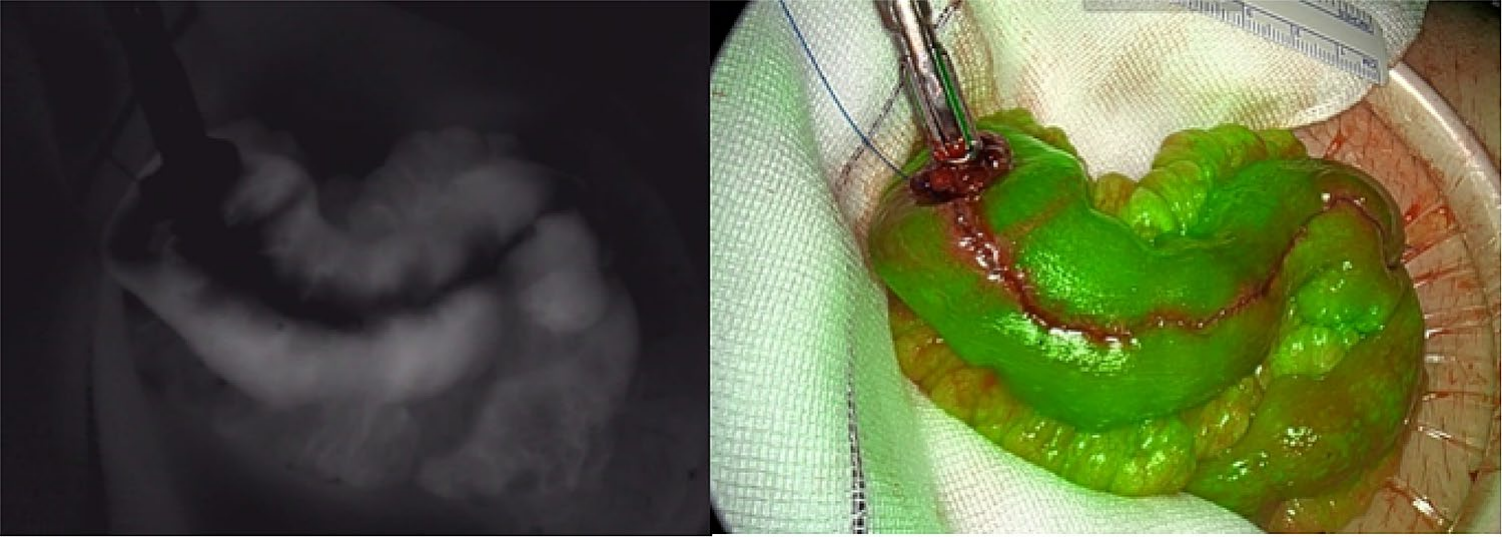
Intraoperative fluorescent and overlay images of pouch perfusion

**Fig. 6:**
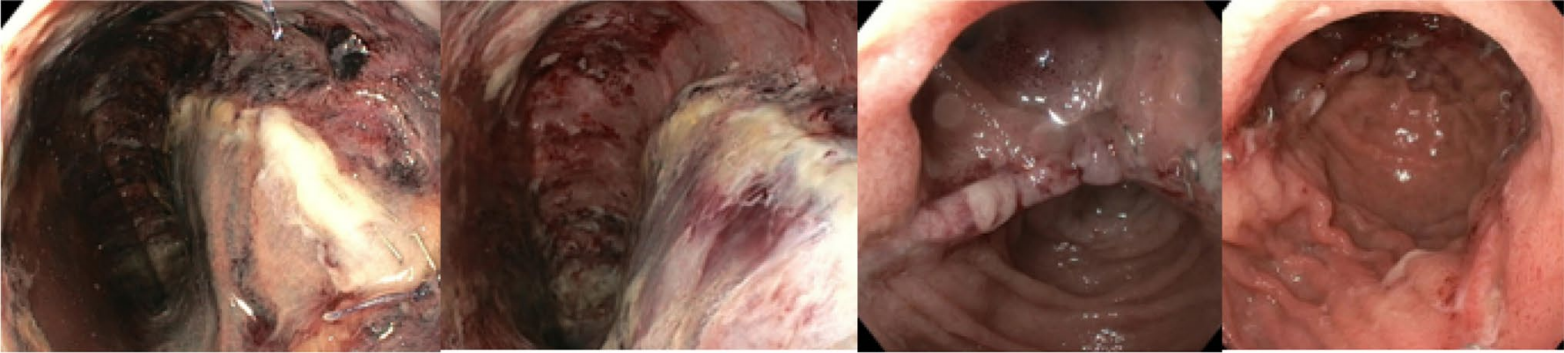
Endoscopy images of the pouch 5 days, 8 days, 3 weeks and 6 weeks postoperatively (portrayed from left to right) showing mucosal ischemia and re-epithelization over time

The second case was a 53-year-old man with a history of biological refractory UC, now in clinical and endoscopic remission under upadacitinib. Because of a sigmoid carcinoma, his immunosuppressants had to be stopped and surgery was indicated. This patient underwent a restorative proctocolectomy and pouch procedure as a planned single-stage procedure. After routine lengthening measures were performed, the reach remained insufficient and additional lengthening measures were indicated. The interconnecting terminal ileal branches were ligated. While the pouch was being constructed, the body of the pouch gradually became discolored (Fig. 4). Subsequent FA showed uniform fluorescent enhancement of the pouch in 33 s (Fig. 5), despite clear discoloration in white light, suggesting venous congestion. Given the FA findings, the pouch was not redone and a double purse-string single-stapled anastomosis was pursued. However, the operative plan was changed from a one-stage to a two-stage procedure. Because of the potential dubious vascularization due to the venous congestion, a pouchoscopy was performed 3 days postoperatively. Extensive mucosal ischemia and sloughing were observed, but the underlying muscular wall seemed well perfused. During repetitive endoscopic follow-up, re-epithelialization of the mucosa was noticed (Fig. 6). No anastomotic leakage occurred and stoma reversal was planned.

## Discussion

During IPAA surgery, it might be necessary to perform lengthening maneuvers that require vessel ligation in order to make the pouch reach [9, 10]. Particularly in these cases, FA might be of value to rule out significant perfusion problems, and even distinguish arterial inflow from venous congestion problems. However, literature describing FA during IPAA is scarce, and change of management after FA has thus far not been recorded [8].

The cases described show that FA was of added value in intraoperative decision-making. In the first case, vascularization of a small area of the pouch body seemed compromised after ligation of both the ileocolic artery and interconnecting terminal ileal branches. FA assessment led to a change of management through suture reinforcement of the hypoperfused area and ileostomy formation. In the second case, even though pouch vascularization in white light seemed severely compromised, fluorescent enhancement occurred in the entire pouch, although in a delayed fashion. Discoloration of the pouch was therefore more likely due to venous congestion. Because of this finding, the surgeons proceeded with the construction of the anastomosis and an uneventful postoperative course followed.

Although there is no agreement in the literature on this subject, FA assessment seems of value if devascularizing measures are required for additional reach. A limitation of this study is the subjective evaluation of the fluorescence images by the surgeon. Quantitative values are warranted to define a threshold for adequate perfusion and venous congestion. Moreover, there is no literature on postoperative outcomes after devascularization to acquire reach without FA assessment. Future comparative studies with larger cohorts are awaited to emphasize the potential added value of FA in pouch surgery, particularly in those cases whereby vessel ligation is indicated to obtain reach.

## Conclusions

ICG FA appears to be of value after ligation of vessels to obtain reach during IPAA.
